# Personalization of CM Injection Protocols in Coronary Computed Tomographic Angiography (People CT Trial)

**DOI:** 10.1155/2020/5407936

**Published:** 2020-01-17

**Authors:** N. G. Eijsvoogel, B. M. F. Hendriks, P. Nelemans, C. Mihl, J. Willigers, B. Martens, J. E. Wildberger, M. Das

**Affiliations:** ^1^Department of Radiology, Maastricht University Medical Center+, P. Debyelaan 25, PO Box 5800, 6202 AZ Maastricht, Netherlands; ^2^CARIM School for Cardiovascular Diseases, Maastricht University Medical Center+, Maastricht, Netherlands; ^3^Department of Epidemiology, Faculty of Health, Medicine and Life Sciences, Maastricht University, Maastricht, Netherlands; ^4^Department of Diagnostic and Interventional Radiology, Helios Kliniken Duisburg, Duisburg, Germany

## Abstract

**Aim:**

To evaluate the performance of three contrast media (CM) injection protocols for cardiac computed tomography angiography (CCTA) based on body weight (BW), lean BW (LBW), and cardiac output (CO). *Materials and methods*. A total of 327 consecutive patients referred for CCTA were randomized into one of the three CM injection protocols, where CM injection was based on either BW (112 patients), LBW (108 patients), or CO (107 patients). LBW and CO were calculated via formulas. All scans were ECG-gated and performed on a third-generation dual-source CT with 70–120 kV (automated tube voltage selection) and 100 kV_qual.ref_/330 mAs_qual.ref_. CM injection protocols were also adapted to scan time and tube voltage. The primary outcome was the proportion of patients with optimal intravascular attenuation (325–500 HU). Secondary outcomes were mean and standard deviation of intravascular attenuation values (HU), contrast-to-noise ratio (CNR), and subjective image quality with a 4-point Likert scale (1 = poor/2 = sufficient/3 = good/4 = excellent). The *t*-test for independent samples was used for pairwise comparisons between groups, and a chi-square test (*χ*2) was used to compare categorical variables between groups. All *p* values were 2-sided, and a *p* < 0.05 was considered statistically significant.

**Results:**

Mean overall HU and CNR were 423 ± 60HU/14 ± 3 (BW), 404 ± 62HU/14 ± 3 (LBW), and 413 ± 63HU/14 ± 3 (CO) with a significant difference between groups BW and LBW (*p*=0.024). The proportion of patients with optimal intravascular attenuation (325–500 HU) was 83.9%, 84.3%, and 86.9% for groups BW, LBW, and CO, respectively, and between-group differences were small and nonsignificant. Mean CNR was diagnostic (≥10) in all groups. The proportion of scans with good-excellent image quality was 94.6%, 86.1%, and 90.7% in the BW, LBW, and CO groups, respectively. The difference between proportions was significant between the BW and LBW groups.

**Conclusion:**

Personalization of CM injection protocols based on BW, LBW, and CO, and scan time and tube voltage in CCTA resulted in low variation between patients in terms of intravascular attenuation and a high proportion of scans with an optimal intravascular attenuation. The results suggest that personalized CM injection protocols based on LBW or CO have no additional benefit when compared with CM injection protocols based on BW.

## 1. Introduction

Cardiac computed tomography angiography (CCTA) is a valuable tool in ruling out coronary artery disease because of its high negative predictive value (99%) [1–4]. Electrocardiogram gated CCTA imaging helps to minimize cardiac motion artifacts [5, 6]. Diagnostic accuracy for coronary artery assessment is achieved with a diagnostic intravascular attenuation (measured in Hounsfield units (HU)) and contrast-to-noise ratio (CNR). Intravascular attenuation depends on scanner settings (e.g., tube voltage or tube potential), and characteristics of the administered contrast media injection protocol (CM) (e.g., concentration, iodine delivery rate (IDR)), and patient-related factors (e.g., cardiac output (CO) and body weight (BW)) [7–9]. IDR, which refers to the grams of iodine injected per second, is the most important factor for achieving diagnostic intravascular attenuation in CCTA [10–12]. Intravascular attenuation should be high enough to assess the coronary arteries (≥325HU) [7]. However, an intravascular attenuation >500 HU could lead to an underestimation of coronary calcifications [13]. Therefore, intravascular attenuation in CCTA should ideally lie within an intravascular attenuation “window” of 325–500HU.

Nowadays, individualization of CM injection protocols based on certain patient parameters is a highly discussed topic. Many different techniques, varying from simple to complicated formulas with differing patient parameters have been suggested, e.g., BW, lean body weight (LBW), and CO [14]. Multiple studies show that individualization based on patients' BW or BW categories results in a diagnostic image quality throughout the patient population, together with decreased CM volumes [15–18].

LBW is a well-known measurement for body composition and is defined as the difference between BW and fatty tissue. Muscle tissue has an increased vascularization compared with fatty tissue [14]. In obese patients, blood volume does not increase linearly to BW. When adapting the CM injection protocol, the possibility of overestimating CM volumes could occur. LBW might be able to adjust for this.

Both blood volume and CO increase according to BW [14]. A higher CO increases the velocity of CM distribution [14], resulting in faster CM bolus arrival and consequently a decreased and shortened intravascular peak attenuation profile compared to a lower CO. Timing and CM volume could be adjusted to CO in order to achieve a similar intravascular attenuation profile between patients with a different CO.

This study addresses the question of whether there are differences in performance between personalized CM injection protocols based on BW, LBW, or CO. The hypothesis is that personalization on either one of these three parameters does not result in a significantly different image quality. Therefore, the aim of this study is to evaluate the performance of three individualized protocols based on BW, LBW, and CO for CCTA.

## 2. Materials and Methods

### 2.1. Ethics and Study Design

The local ethical committee and institutional review board approved the study design (METC number: 16-1-110). This single center randomized controlled trial is registered on Clinicaltrials.gov under reference number NCT03292354.

All patients referred for a standard CCTA were eligible for inclusion. Patients were included after obtaining informed consent. Exclusion criteria were hemodynamic instability, pregnancy, renal insufficiency (GFR <30 mL/min/1.73 m^2^), iodine allergy, age <18 years, and/or no informed consent. All clinically available scan protocols and tube voltages (70–120 kV) were included to simulate daily clinical practice. All patients were weighed just before scanning. After obtaining written informed consent, patients were randomized into one of three study groups: BW, LBW, or CO. Patient characteristics (i.e., age, height, BW, body mass index (BMI), LBW, and CO) and scan indication were recorded.

**Figure 1:**
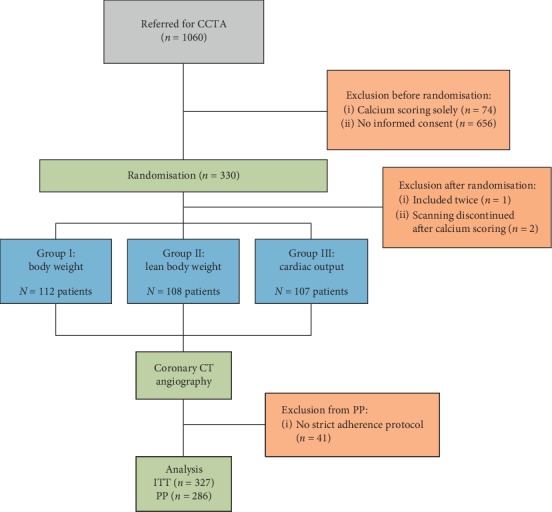
Flowchart.

Figure 1 shows the flowchart of the study design.

### 2.2. Randomization

Patients were randomized into three groups in a 1 : 1 : 1 ratio by a dedicated software (ALEA Clinical B.V., FormsVision, Abcoude, the Netherlands). Randomization was stratified on age (<50 yr; 50–69 yr; >70 yr) and weight (<60 kg; 60–69 kg; 70–90 kg; >90 kg) categories. Variable random permuted blocks were used to conceal treatment allocation.

### 2.3. Scan Protocol

Patients could be scanned with three CCTA protocols, based on their heart rate. The scan protocols are depicted in Table 1. In case of a high heart rate (>70 bpm), preparation consisted of oral beta blockade (50 mg metoprolol tartrate) two hours prior to scanning, or intravenous beta blockade (5–20 mg metoprolol tartrate) just before scanning. Furthermore, sublingual nitroglycerin (Nitrolingual pump spray, Isordil®, Pohl-Boskamp, Hohenlockstedt, Germany) was administered prior to scanning in order to increase visualization of the coronary arteries. All scans were performed on a 3^rd^ generation dual-source CT scanner (Somatom Definition Force, Siemens Healthineers, Forchheim, Germany).

All dose-related parameters were recorded with dose monitoring software (Radimetrics Enterprise Platform™, Bayer Healthcare, Berlin, Germany).

### 2.4. Injection Protocol

An 18–22 gauge intravenous injection catheter was inserted in either left or right antecubital vein for CM administration. For flow rates over 7 mL/s, a special 18 gauge needle (BD Nexiva Diffusics® I.V. Catheters, Sandy, UT, USA) was inserted [19]. This needle has three additional tear-drop shaped diffusion exit points close to the tip and a strengthened design that enables use with power injectors set up to 325 psi. The CM concentration was 300 mgI/mL (Iopromide, Bayer), prewarmed to body temperature (37°C; 98.6°F) and injected with help of a dual-head CT power injector (Stellant, Bayer).

Patients were randomized and enrolled in one of three CM injection protocols (Group 1: group BW; Group 2: group LBW; Group 3: group CO) with tailored flow rates and consequtively tailored IDRs. The injection protocols were defined with help of a formula, which has been explained extensively elsewhere [20] and can be found in Appendix 1. Besides personalization to these patient characteristics, the injection protocol was adapted to the tube voltage, as described in previous studies [21–25].

The following formulas were used:

(i) BW: flow rate = 0.0007976 ^*∗*^ kV ^*∗*^ BW
 With BW in kg
(ii) LBW: Flow rate = 0.001101 *∗* kV *∗* LBW
  - LBW according to the Hume formula [26]
  -  Man: LBW = 0.32810 *∗* BW + 0.33929 *∗L* – 29.5336
  -  Woman: LBW = 0.29569 *∗* BW + 0.41813 *∗L* –43.2933
  -   With BW in kg and *L* = length in cm
(iii) CO: flow rate = 0.008916 *∗* kV*∗* CO 
  (b) CO according to an adapted Katori formula [27]
     CO = (4.874 − 0.023 *∗* age) *∗* BSA
      With age in years and BSA = body surface area

The formulas shown above determined the flow rates for both test bolus and main bolus. The injection time was fixed for the test bolus (2 s) and the three scan protocols: 8 s for flash and 10 s for adaptive sequence (AS) and helical. The CM volume of the test bolus and main bolus (mL) was then derived by multiplying the calculated flow rate (mL/s) with the injection time (s).

All relevant CM injection parameters were recorded by CM monitoring software (Certegra™, Bayer). The total iodine load (TIL) and IDR were calculated additionally.

### 2.5. Data Analysis

All CT images were analyzed with multiplanar reconstructions and axial slices on dedicated software (Syngo.Via™, Siemens). The 17-segment model of the American Heart Association (AHA) was used to assess the segments of all coronary arteries [28]. All outcomes were recorded on a per-patient and on per-segment level. Assessment of coronary artery calcifications and stenosis was not within the scope of this article. However, Agatston score and calcium mass were collected from the radiology reports and evaluated between groups.

#### 2.5.1. Image Analysis: Primary Outcome

The primary outcome was defined as the proportion of scans with an intravascular attenuation between 325–500 HU. An intravascular attenuation of ≥325 HU was considered diagnostic [7, 29]. An intravascular attenuation >500 HU was considered unnecessarily high [13]. The intravascular attenuation was measured by one researcher, trained for objective assessment and blinded for treatment allocation (N. E.). Automatically drawn centerlines in all three coronary arteries were used. Circular regions of interest (ROIs) were manually drawn in the coronary arteries and epicardial fat to measure intravascular attenuation in HU and image noise (defined as the standard deviation of the intravascular attenuation). The epicardial fat measurements were used to calculate CNR and signal-to-noise ratio (SNR) (see *secondary outcomes*). The overall mean (per-patient) intravascular attenuation was defined as the mean of the intravascular attenuation of all present segments. The circumference of the ROIs was kept as large as possible (minimal surface 1 mm^2^) whilst avoiding arterial walls, intravascular plaques, and stents. In case of a CABG, all segments with an assessable lumen were used to draw ROIs.

#### 2.5.2. Image Analysis: Secondary Outcome

CNR was determined as intravascular attenuation minus epicardial fat attenuation, divided by the standard deviation of the epicardial fat attenuation. Intravascular attenuation divided by the standard deviation of the intravascular attenuation was used to determine SNR [18, 21, 30].

Both per-patient and per-segment subjective image qualities were assessed by the same, blinded, researcher by using a 4-point Likert scale: Poor—major artifacts and/or low intravascular attenuation; Sufficient—minor artifacts and/or insufficient intravascular attenuation, however still diagnostic for coronary assessment; Good—minimal artifacts and sufficient intravascular attenuation; and Excellent—no artifacts and sufficient intravascular attenuation.

### 2.6. Statistical Analysis

IBM SPSS software (version 23.0, IBM SPSS statistics, Chicago IL, USA) software was used for statistical analysis. Continuous variables are expressed as mean ± standard deviation and categorical variables as absolute numbers and percentages (%). Both intention-to-treat (ITT) and per-protocol (PP) analyses were performed to assess the robustness of the results. The ITT analysis included randomized patients for whom outcome data were available. The PP analysis included a subset of patients with strict adherence to the assigned CM injection protocol. A chi-square test (*χ*^2^) was used to compare categorical variables between groups. The *t*-test for independent samples was used for pairwise comparisons of continuous variables between groups. All *p* values were 2-sided, and a *p* < 0.05 was considered statistically significant.

## 3. Results

### 3.1. Patient Characteristics

A total of 330 patients were randomly assigned to one of the three experimental study arms between April 2017 and July 2018. Three patients were excluded after randomization; one patient was randomized twice due to randomization software problems, and two patients were excluded because scanning was discontinued after the calcium scoring. Thus, a total of 327 patients remained for the ITT analysis. A total of 41 patients without strict adherence to the assigned CM injection protocol were excluded from the PP analysis (*n* = 286). These patients (group BW *n* = 13; group LBW *n* = 17; and group CO *n* = 11) received a different protocol for various reasons, e.g., flow rate had to be adjusted because it was not applicable to the needle size.

Randomization placed 112 patients in group BW, 108 patients in group LBW, and 107 patients in group CO. The distribution of baseline characteristics was similar between groups (Table 2).

#### 3.2. CM Dose

All injection protocol parameters can be found in Table 3. Mean main bolus volume was 47.5 ± 17.8 mL for group BW, 44.7 ± 13.9 mL for group LBW, and 42.7 ± 11.5 mL for group CO. The mean flow rate for groups BW, LBW, and CO was 5.1 ± 1.7 mL/s, 4.8 ± 1.2 mL/s, and 4.6 ± 1.0 mL/s, respectively. Although small differences existed in injection parameters between groups, a significant difference was found in all injection parameters between group BW and group CO, but not between the other groups.

### 3.3. Image Analysis

#### 3.3.1. Attenuation Parameters: Patient Level

**Figure 2:**
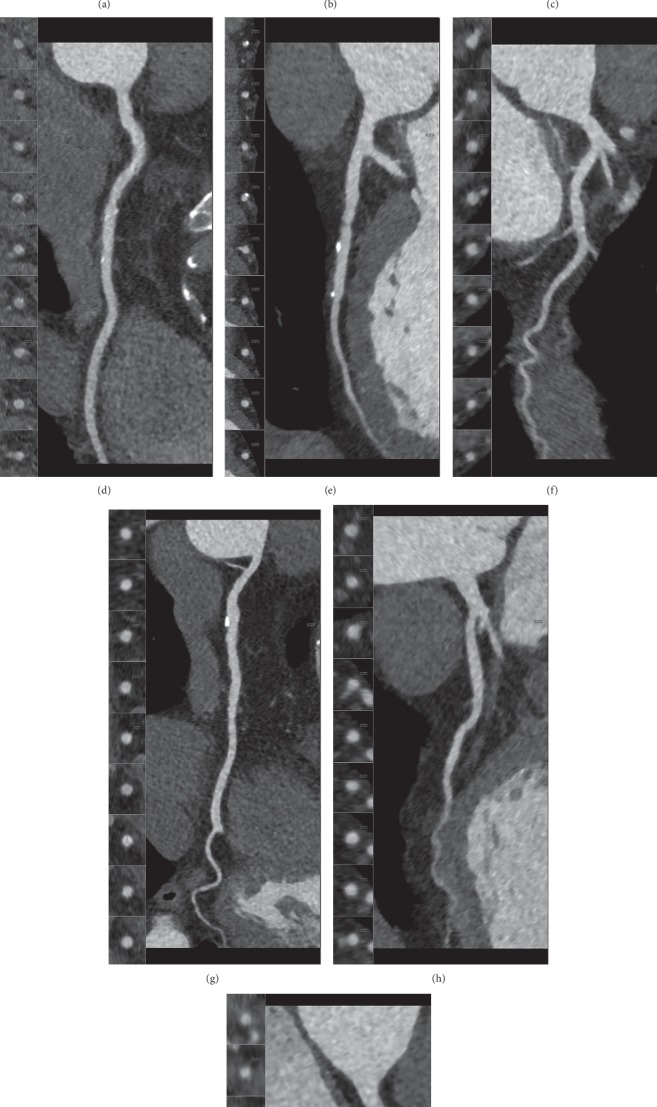
Three different scans with a Likert scoring of 4. (a, b, c) RCA, LAD, and Cx in a patient scanned with the BW protocol. (d, e, f) RCA, LAD, and Cx in a patient scanned with the LBW protocol. (g, h, i) RCA, LAD, and Cx in a patient scanned with the CO protocol. Attenuation is homogeneous between all three protocols.

All image quality parameters on a per-patient and per-segment level are presented in Table 4. The proportion of patients with an optimal intravascular attenuation predefined as 325–500 HU was 83.9% in group BW, 84.3% in group LBW, and 86.9% in group CO. No significant differences in proportions were found between the groups (Figure 2).

**Figure 3:**
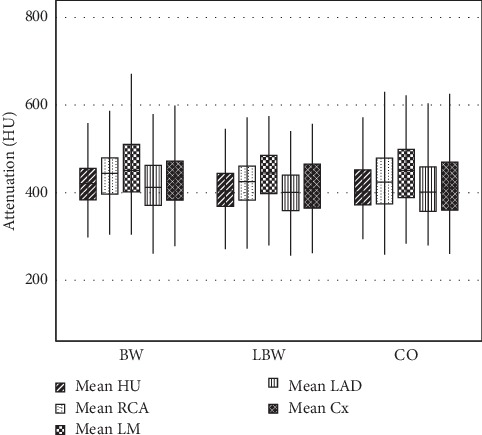
Mean overall attenuation and mean attenuation for the right coronary artery (RCA), left main (LM), left anterior descending (LAD), and circumflex artery (Cx). Group CO resulted in an increased number of scans between 325 and 500HU. However, group BW had less scans <325 HU. All these differences were not significantly different. Of these three, group LBW had a lower attenuation and less scans >325 HU.

The difference in mean intravascular attenuation between groups BW and LBW was significant (*p*=0.024), but not between the other pairwise comparisons (Table 4). The mean intravascular attenuation was diagnostic (≥325 HU) in 95.5% of the individuals in the BW group, 89.8% in the LBW group, and 95.3% in the CO group (Figure 3 and Table 4). No significant differences in these proportions were found between the groups. In one case in the LBW group, the intravascular attenuation was <325 HU due to an occlusion in the left subclavian vein. In the other cases, it was due to timing and/or movement artifacts.

#### 3.3.2. Subjective Image Quality: Patient Level

**Figure 4:**
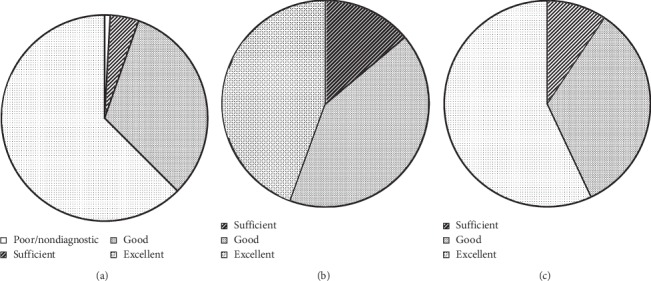
Likert score distribution in the four groups. A significant difference was found in Likert score between group BW and group LBW (*p*=0.031), but not between other groups. ^*∗*^Poor image quality in one case scanned with a “helical” protocol due to severe motion artifacts.

In group BW, the proportion of scans with good-excellent image quality was 94.6%; in the LBW group, this proportion was 86.1%; and in the CO group, it was 90.7%. Pairwise comparisons between all groups showed a significant difference between groups BW and LBW (*p*=0.031) but not between the other groups (Table 4). One scan in the BW group, scanned with a helical protocol, was graded nondiagnostic due to extensive motion artifacts (Figure 4). Subjective image quality on a per-segment level can be found in Table 4.

#### 3.3.3. Per-Protocol Analysis

The results of the PP analysis excluding 41 patients without strict adherence to the assigned CM injection protocol are presented in Appendix 2 and Appendix 3. These results are similar to those from the ITT analysis.

#### 3.3.4. Radiation Dose

All information on scan protocol and tube voltage selection is depicted in Table 2. The complete dose report can be found in Table 5.

## 4. Discussion

Three patient parameters (BW, LBW, and CO) were used to create tailored CM injection protocols in CCTA scanning. The use of different patient characteristics for tailoring CM injection protocols resulted in only minor differences in mean intravascular attenuation, with the highest attenuation found in the BW group. With regard to proportion of scans between 325–500 HU and proportion of diagnostic scans (mean overall intravascular attenuation ≥325 HU), no significant between-group differences were found. However, the higher mean intravascular attenuation value in group BW (compared to the LBW) did result in significantly higher proportions of scans with good to excellent image quality, which can be considered clinically relevant.

Previously, the CM injection protocol in our institution was based on BW categories (i.e., <50 kg, 50–69 kg, 70–90 kg, and >90 kg). This protocol resulted in a higher mean intravascular attenuation with a relatively wide standard deviation (464 ± 85 HU). There was a very wide range (186–672 HU) in intravascular attenuation values and an optimal intravascular attenuation between 325–500 HU was observed in 56.4% of patients. The CM injection protocols in the current study resulted in less variation in intravascular attenuation values between patients with standard deviation values around 60 HU and higher proportions of scans within the optimal window of 325–500 HU.

All CM injection parameters between groups BW and CO were significantly different. Group BW used a higher CM volume of 5 mL compared with group CO. This increase of 5 mL did not lead to a significant difference in intravascular attenuation nor to significantly more scans. Furthermore, the CM volume between groups LBW and BW did not differ significantly, although a significantly lower attenuation was found in group LBW. However, absence of a significant difference does not mean no difference is present [31]. It is possible that all small differences in patient parameters and injection protocol parameters resulted in the significant difference in attenuation. The current study shows that the total CM volume used does not have to be the deciding factor in selecting an individualized CM injection protocol. These results also show that individualization to BW works equally well as more intricate patient parameters and formulas.

The use of BW and LBW in personalization of CM injection protocols has been studied before in varying degrees. Liu et al. showed that BW-adapted injection protocols are feasible in high-pitch prospective CCTA [32]. Liu et al. did not adapt the scan protocol to the individual patient, and they only researched high-pitch CCTA scanning, whereas the current study used all currently available CCTA scan protocol techniques. Contrary to Liu et al., Tatsugami et al. did adapt their scan protocol to BMI and showed similar results to the current study [33]. In the current study, scan protocol adaptation was performed by automated tube voltage software (ATVS). This may be more specific compared to only using patients' BMI, as the addition of ATVS takes multiple other factors into account; not only body habitus but also scan protocol is taken into account. Furthermore, a study conducted by Bae et al. researched different patient parameters. They stipulated that BW is the most important patient-related factor for intravascular attenuation [7]. Only one study investigated the use of LBW in CTA of the aorta [34]. This study did not find any significant differences in intravascular attenuation between LBW and BW, which is different from the findings in the current study. Lastly, to our knowledge, this is the first study to individualize the injection protocols to the patients' CO.

The current study has some limitations. Due to lack of correlation with invasive angiography, it could not be verified whether a higher proportion of patients with optimal intravascular attenuation (325–500 HU) resulted in a more accurate diagnosis and decreased underestimation of calcifications. Second, in the current study, CO was calculated with the help of a formula, and CO measurement was not performed.

To conclude, personalization of CM injection protocols to BW, LBW, and CO, and scan time and tube voltage in CCTA resulted in low variation in intravascular attenuation between patients in the three groups and a high proportion of scans with an optimal intravascular attenuation. The results suggest that personalized CM injection protocols based on LBW or CO have no additional benefit when compared to CM injection protocols based on BW.

## Figures and Tables

**Table 1:** Scan parameters for the used scan protocols.

Parameter	High-pitch “flash” protocol	Adaptive sequence “AS” protocol	Helical protocol
Scout scans			
Tube voltage	100 kV	100 kV	100 kV
Tube current	30 mAs	30 mAs	30 mAs

Calcium scoring			
Tube voltage	120 kV	120 kV	120 kV
Tube current	80 mAs	80 mAs	80 mAs

CCTA			
Heart rate	≤70 bpm	70–90 bpm	>90 bpm
Tube voltage	CARE kV	CARE kV	CARE kV
Qual. ref. tube voltage	100 kV_qual.ref_	100 kV_qual.ref_	100 kV_qual.ref_
Qual. ref. tube current	300 mAs_qual.ref_	300 mAs_qual.ref_	300 mAs_qual.ref_
Slider position	11	11	11
Delay	Test bolus	Test bolus	Test bolus
Collimation	2 *∗* 192 *∗* 0.6 mm	2 *∗* 160 *∗* 0.6 mm	2 *∗* 192 *∗* 0.6 mm
Rotation time	0.25 s	1.25 s	0.25 s
Pitch	3.2	—	0.15

Image reconstruction			
Slice thickness	0.6 mm	0.6 mm	0.6 mm
Increment	0.4 mm	0.4 mm	0.4 mm
Kernel	Bv40	Bv36	Bv40
Strength	2	3	3

Bpm = beats per minute; CCTA = coronary cardiac computed tomography; Qual. ref. = quality reference.

**Table 2:** Baseline characteristics between groups.

Parameter	Group BW (*n* = 112)	Group LBW (*n* = 108)	Group CO (*n* = 107)
Age (years)	60.0 ± 10.1	59.2 ± 10.1	59.5 ± 11.7

Sex			
Women (*n*%)	56 (50)	54 (50)	58 (54.2)
Men (*n*%)	56 (50)	54 (50)	49 (45.8)

Height (m)	1.73 ± 0.1	1.73 ± 0.1	1.73 ± 0.1

Weight (kg)	83.1 ± 16.7	83.6 ± 15.4	83.2 ± 15.5

BMI (kg/m^2^)	27.9 ± 5.0	27.9 ± 4.2	27.8 ± 4.9

BSA (m^2^)	2.0 ± 0.2	2.0 ± 0.2	2.0 ± 0.2

CO (L/min)	6.8 ± 1.0	6.9 ± 0.9	6.9 ± 0.9

LBW (kg)	54.8 ± 8.7	55.0 ± 9.0	54.7 ± 8.0

HR (bpm)	65.0 ± 14.7	64.9 ± 11.6	66.5 ± 12.9

Indication scan (*n* %)			
Atypical chest pain	50 (44.6)	59 (54.6)	52 (48.6)
Typical chest pain	7 (6.3)	4 (3.7)	3 (2.8)
CAD	33 (29.5)	29 (26.9)	38 (35.5)
AF	8 (7.1)	7 (6.5)	4 (3.7)
Other	14 (12.5)	8 (7.4)	10 (9.3)
Not available	—	1 (0.9)	—

Preparation (*n*%)			
Nitroglycerin	109 (97.3)	107 (99.1)	101 (94.4)
Beta-blocker	8 (7.1)	9 (8.3)	14 (13.1)
Selokeen (i.v.)	7 (6.3)	3 (2.8)	7 (6.5)

Scan protocol (n%)			
Flash	79 (70.5)	70 (64.8)	71 (66.4)
Adaptive sequence	26 (23.2)	29 (26.9)	31 (29.0)
Helical	7 (6.3)	9 (8.3)	5 (4.7)

Tube voltage (kV) *n* (%)			
70	44 (39.3)	41 (38.0)	46 (43.0)
80	42 (37.5)	45 (41.7)	39 (36.4)
90	10 (8.9)	12 (11.1)	11 (10.3)
100	5 (4.5)	4 (3.7)	7 (6.5)
110	—	—	—
120	11 (9.8)	6 (5.6)	4 (3.7)
Agatston score	176 ± 417	178 ± 344	147 ± 410
Calcium mass (equivalent mass/mg)	31 ± 72	33 ± 63	28 ± 75

*Note*. Values are presented as mean ± standard deviation or numbers. BW = body weight; LBW = lean body weight; CO= Cardiac output; BMI = body mass index; BSA = body surface area; HR = heart rate; CAD = coronary artery disease; AF = atrial fibrillation; i.v. = intravenous.

**Table 3:** Resulting injection parameters for the different groups.

	Group BW (*n* = 112)	Group LBW (*n* = 108)	Group CO (*n* = 107)	*p* value BW vs. LBW	*p* value BW vs. CO	*p* value LBW vs. CO
CM volume (mL)	47.5 ± 17.8 (18.4–100.2)	44.7 ± 13.9 (26.2–105.9)	42.7 ± 11.5 (27.0–101.3)	0.190	0.019^*∗*^	0.271
Test bolus (mL)	11.2 ± 4.0 (4.1–23.0)	10.3 ± 2.8 (6.0–20.9)	9.8 ± 2.2 (6.8–17.0)	0.047	0.002^*∗*^	0.192
Flow rate (mL/s)	5.1 ± 1.7 (2.1–9.1)	4.8 ± 1.2 (2.9–8.5)	4.6 ± 1.0 (3.2–9.1)	0.097	0.010^*∗*^	0.280
IDR (gI/s)	1.5 ± 0.5 (0.6–2.7)	1.4 ± 0.4 (0.9–2.6)	1.4 ± 0.3 (1.0–2.7)	0.085	0.008^*∗*^	0.306
TIL (gI)	14.3 ± 5.3 (5.5–30.1)	13.4 ± 4.2 (7.9–31.8)	12.8 ± 3.5 (8.1–30.4)	0.192	0.020^*∗*^	0.273
Peak pressure (psi)	111.5 ± 48.1 (30.0–262.0)	105.2 ± 39.6 (41.0–248.0)	98.9 ± 34.3 (52.0–275.0)	0.290	0.026^*∗*^	0.218

*Note*. Values are presented as mean ± standard deviation and ranges or numbers (percentages). BW = body weight; LBW = lean body weight; CO = cardiac output; CM = contrast media; IDR = iodine delivery rate; TIL = total iodine load. ^*∗*^A significant difference was found between the group BW and group CO.

**Table 4:** Primary and secondary outcomes for all groups on a per-patient and per-segment level.

Parameter	Group BW	Group LBW	Group CO	*p* value BW vs. LBW	*p* value BW vs. CO	*p* value LBW vs. CO
No. of patients	*n* = 112	*n* = 108	*n* = 107			
Image quality parameters per patient						
Attenuation (HU)	423 ± 60	404 ± 61	413 ± 63	0.024^*∗*^	0.211	0.333
Scans 325–500HU (%)	83.9	84.3	86.9	0.947	0.532	0.579
Scans ≥325HU (%)	95.5	89.8	95.3	0.102	0.941	0.124
Noise	39 ± 8	39 ± 8	40 ± 9	0.844	0.332	0.451
CNR	14 ± 3	14 ± 3	14 ± 3	0.313	0.094	0.530
SNR	12 ± 3	11 ± 3	12 ± 5	0.117	0.970	0.265
Good-excellent image quality (%)	94.6	86.1	90.7	0.031^*∗*^	0.257	0.299

No. of segments	*n* = 1.599	*n* = 1.556	*n* = 1.523			
Image quality parameters per segment						
Scans 325–500HU (%)	67.4	67.4	66.5	1.000	0.618	0.621
Scans ≥325HU (%)	90.7	87.2	88.6	0.002^*∗*^	0.061^†^	0.224
Good-excellent image quality (%)	82.9	75.1	78.6	<0.001^*∗*^	0.003^†^	0.023^•^

*Note*. Values are presented as mean ± standard deviation. BW = body weight; LBW = lean body weight; CO = cardiac output; HU= Hounsfield units; CNR = contrast-to-noise ratio; SNR = signal-to-noise ratio. ^*∗*^A significant difference was found between group BW and group LBW. ^†^A significant difference was found between group BW and group CO. ^•^A significant difference was found between group LBW and group CO.

**Table 5:** Dose report for different scan protocols and groups.

Parameter	Flash	AS	Helical
Effective tube current (mAs)^*∗*^			
Group BW	494 ± 81	354 ± 63	430 ± 99
Group LBW	511 ± 79	357 ± 80	454 ± 80
Group CO	508 ± 74	352 ± 62	405 ± 113

CTDI_vol_ (mGy)^*∗*^			
Group BW	2.5 ± 1.4	19.8 ± 15.7	42.0 ± 34.7
Group LBW	2.5 ± 1.3	17.1 ± 13.1	42.7 ± 17.5
Group CO	2.3 ± 1.0	22.2 ± 28.9	30.9 ± 22.9

Total DLP (mGy ^*∗*^ cm)^*∗*^			
Group BW	81 ± 31	290 ± 198	664 ± 471
Group LBW	78 ± 27	273 ± 195	730 ± 272
Group CO	74 ± 25	271 ± 245	521 ± 385

Effective dose (mSv)^*∗*^			
Group BW	0.9 ± 0.5	5.4 ± 3.7	15.4 ± 12.1
Group LBW	0.8 ± 0.4	6.0 ± 4.9	11.8 ± 3.1
Group CO	0.8 ± 0.4	5.2 ± 4.5	9.9 ± 7.4

*Note*. Values are presented as mean ± standard deviation. Flash = high-pitch protocol; AS = adaptive sequence protocol; Helical = helical protocol. BW = body weight; LBW = lean body weight; CO = cardiac output; CTDI_vol_ = CT dose index; DLP = dose length product. ^*∗*^No significant difference was found in all radiation dose parameters between all groups (all *p* > 0.100).

## Data Availability

For ethical and legal reasons, the data that we collected cannot be made publicly available. Firstly, the study was approved by the Medical Ethics Committee (METC) of the Maastricht University Medical Center, Maastricht, the Netherlands, under the condition that access to the data is granted only to (1) members of the research team, (2) the Medical Ethics Committee members that approved this study, and (3) authorized personnel of the Health Care Inspectorate. Secondly, participants did not consent to publicly archiving their data. However, requests for an anonymized dataset can be sent to data management group at Clinical Trial Center Maastricht at datamanagement.ctcm@mumc.nl where the data are stored under the METC reference number: 16-1-110.

## Supplementary Materials

Supplementary Materials Appendix 1: development and definition of the different injection protocol formulas. Appendix 2: all injection parameters for the individual groups for the per-protocol analysis. Appendix 3: primary and secondary outcomes for all groups on a per-patient and per-segment level for the per-protocol analysis.
