# Supplementary material for: Personalization of CM Injection Protocols in Coronary Computed Tomographic Angiography (People CT Trial)
Source: Contrast Media Mol Imaging. 2020 Jan 17;2020:5407936. doi: 10.1155/2020/5407936 (PMC7201621; doi:10.1155/2020/5407936)
Supplement: Supplementary Materials — Appendix 1: development and definition of the different injection protocol formulas. Appendix 2: all injection parameters for the individual groups for the per-protocol analysis. Appendix 3: primary and secondary outcomes for all groups on a per-patient and per-segment level for the per-protocol analysis. [file 5407936.f1.pdf]

Appendix 1.

The formula was first developed to be able to calculate the concentration of contrast media (CM) in the blood of the patient (c; mgI/mL). To be able to predict the concentration of the CM in the blood, the amount of CM that remains in the heart (or mixes in the heart) has to be taken into account. The following formula accounts for the flow rate of the CM injected (F; mL/s), the concentration of the CM used (Ic; mgI/mL), the CO (O; mL/s), the volume of CM that remains in the heart (V; mL), the time of injection (t; s) and the patient factor for patient variability ($e^{-(o/v)*t}$):

$$c = \frac{F * I_c}{O}(1 - e^{-\frac{O}{V}*t})$$

This formula was then adapted to be able to account for an sufficient intravascular attenuation. The known relation between intravascular attenuation (HU) and concentration of contrast in the blood (HU=k*c; with k being a constant) [8; 14] and the known inverse relation between HU and kV setting (HU=1/kV) [25] was used to develop a formula which calculates the expected attenuation in a scan. A reference kV setting of 120kV was used, and the following formula was developed:

$$HU^{kVp} = k * \frac{F * I_c}{kVp} * patient\ factor$$

In this formula, cardiac output (CO), lean body weight (LBW) and body weight (BW) were inserted as patient factor. The formulas for these parameters are taken from previous studies [26; 27]. To be able to rewrite the formula and calculate the iodine delivery rate (IDR), flow rate and CM volume for the individual patient, a mean 'reference patient' was established with help of previous research [34]. This 'reference' patient was a man, 56 years old, length 1.71m, weight 75kg, scanned with 100kV and flow rate of 6.0mL/s CM (300mgI/mL Iopromide). The reference patient was applied to the formula with the different given parameters. The formula was then transformed into the formulas shown below.

- BW (group 1): Flow rate=0.0007976*kV*BW;
    - With BW in kg.
- LBW (group 2): Flow rate=0.001101*kV*LBW. LBW (Hume formula)
    - Man: LBW=0.32810*BW+0.33929*L–29.5336 [26]
    - Woman: LBW=0.29569*BW+0.41813*L–43.2933 [26]

        - With BW in kg and L=length in cm;

- CO (group 3): Flow rate=0.008916*CO*kV;
    - CO = (4,874-0,023*yr)*BSA [27]. CO: Katori formula
        - With BSA = Body surface area;

Appendix 2.

Resulting injection parameters for the different groups.

	Group BW (n = 99)	Group LBW (n = 91)	Group CO (n = 96)	*p*-value BW vs. LBW	*p*-value BW vs. CO	*p*-value LBW vs. CO
CM volume (mL)	46.6±16.9 [22.1-100.2]	43.4±11.9 [26.2-89.0]	42.2±11.6 [27.1-101.3]	0.132	0.038*	0.506
Test bolus (mL)	11.0±3.7 [6.0-21.9]	10.1±2.6 [6.0-18.0]	9.8±2.2 [6.8-17.0]	0.072	0.007*	0.312
Flow rate (mL/s)	5.0±1.6 [2.7-9.1]	4.7±1.1 [2.9-8.2]	4.6±1.0 [3.2-9.1]	0.113	0.023*	0.429
IDR (gI/s)	1.5±0.5 [0.8-2.7]	1.4±0.3 [0.9-2.5]	1.4±0.3 [1.0-2.7]	0.100	0.021*	0.468
TIL (gI)	14.0±5.1 [6.6-30.1]	13.0±3.6 [7.9-26.7]	12.7±3.5 [8.1-30.4]	0.133	0.039*	0.510
Peak pressure (psi)	107.8±43.5 [41.0-229.0]	103.5±38.7 [41.0-248.0]	99.0±35.1 [52.0-275.0]	0.469	0.120	0.407

Note – Values are presented as means±standard deviation and [ranges] or numbers (percentages).

BW = body weight; LBW = lean body weight; CO = cardiac output; CM = contrast media; IDR = iodine delivery rate; TIL = total iodine load.

*A significant difference was found between group BW and group CO.

Appendix 3.

Primary and secondary outcomes for all groups on a per-patient and per-segment level.						
Parameter	Group BW	Group LBW	Group CO	*p*-value<br>BW vs. LBW	*p*-value<br>BW vs. CO	*p*-value<br>LBW vs. CO
No. of patients	n=99	n=91	n=96			
Image quality parameters per patient						
Attenuation (HU)	425±55	401±59	413±58	0.005*	0.149	0.158
Scans 325-500HU (%)	86.9	86.8	91.7	0.991	0.280	0.283
Scans ≥325HU (%)	97.0	90.1	97.9	0.052	0.676	0.023•
Noise	39±8	39±7	40±9	0.777	0.295	0.197
CNR	14±3	14±3	13±3	0.599	0.092	0.280
SNR	12±3	11±3	12±5	0.109	0.927	0.252
Good-excellent image quality (%)	93.9	86.8	92.7	0.094	0.730	0.182
No. of segments	n=1.417	n=1.301	n=1.367			
Image quality parameters per segment						
Scans 325-500HU (%)	68.7	72.1	66.4	0.117	0.387	0.016•
Scans ≥325HU (%)	91.5	89.2	85.9	<0.001*	0.007†	0.041•
Good-excellent image quality (%)	83.1	76.3	79.7	<0.001*	0.024†	0.033•

- *Note: Values are presented as means±standard deviation.*

BW= body weight; LBW= lean body weight; CO= cardiac output; HU= Hounsfield units; CNR= contrast-to-noise ratio; SNR= signal-to-noise ratio.

*A significant difference was found between group BW and group LBW.

† A significant difference was found between group BW and group CO.

• A significant difference was found between group LBW and group CO.
